# Elucidating metabolites and biosynthetic pathways during musk maturation: insights from forest musk deer

**DOI:** 10.3389/fphar.2025.1503138

**Published:** 2025-04-28

**Authors:** Hang Jie, Feng Li, Qian Liu, Tingting Zheng, Helin Tan, Xiaolan Feng, Guijun Zhao, Dejun Zeng, Diyan Li, Zhongxian Xu, Tao Wang

**Affiliations:** ^1^ Sichuan Wildlife Rehabilitation and Breeding Research Center, Key Laboratory of Southwest China Wildlife Resources Conservation (Ministry of Education), China West Normal University, Nanchong, China; ^2^ Bio-resource Research and Utilization Joint Key Laboratory of Sichuan and Chongqing, Chongqing Institute of Medicinal Plant Cultivation, Nanchuan, Chongqing, China; ^3^ Antibiotics Research and Re-evaluation Key Laboratory of Sichuan Province, School of Pharmacy, Sichuan Industrial Institute of Antibiotics, Chengdu University, Chengdu, China

**Keywords:** forest musk deer (*Moschus berezovskii* Flerov), musk maturation, metabolites, biosynthesis, pathway

## Abstract

**Background:**

Musk is a blackish-brown solid used in traditional Chinese medicine with a unique and intense scent. Limited evidence on its function and pathways is available from databases due to the complexity, variability, and derivativity of chemical composition.

**Results:**

In this study, musk samples from three different stages during maturation: the end of June (group A), August (group B), and October (group C) were harvested from six male forest musk deer. A gas chromatography and mass spectrometry (GC-MS) approach was used to explore the chemical composition. Results indicated the presence of 66 known and 14 unknown chemicals, including 29 aromatic compounds. Lipids (51.52%), organic oxygen compounds (28.79%), and organoheterocyclic compounds (12.12%) were the most abundant substances. A total of 13 differential metabolites were found, including four macrocyclic ketones and six androgens and derivatives that increased as musk matured. Biosynthesis of unsaturated fatty acids was enriched in differential metabolites across stages. Tetracosanoic acid, methyl ester, and TES1 [EC: 3.1.2.2] participated in the biosynthesis of muscone. A total of nine chemicals and six steroidogenic enzymes participated in steroid hormone biosynthesis.

**Conclusion:**

This study annotates and defines metabolites in musk systematically, macrocyclic ketones (9.09%) and lipids (51.52%) were categorized unambiguously, suggesting that previous studies have underestimated the lipid content in musk, and critical role for lipid metabolism in musk gland development and odor profile formation. The high lipid content may reflect energy storage for glandular activity or serve as precursors for volatile compound synthesis, offering new mechanistic insights into musk maturation. Therefore, we preliminarily decipher the biosynthetic pathways of muscone and steroids through providing involved enzymes and metabolites. These results will deepen the understanding of the composition of natural musk and offer new theoretical insights to promote the comprehensive use of this resource.

## Background

Forest musk deer (*Moschus berezovskii* Flerov), a small ruminant in south-central China and northern Vietnam, has been listed as an endangered species by the International Union for Conservation of Nature (IUCN) due to habitat loss and poaching for musk sac (Yang et al., 2003; Geng and Ma, 2000). Musk, produced by the musk gland surrounding the male deer’s prepuce (Jie et al., 2014), is valued in traditional Chinese medicine (TCM), with high medicinal and economic value (Pharmacopoeia Commission, 2020; Meng et al., 2012). Rich scent chemistry and recognition of volatile compounds have been applied to scent gland marking and social behavior by animals (Thiessen and Rice, 1976; Ehlers and Schulz, 2023; Zhou et al., 2021). Deep brown mature musk has unique and intense scents, attracting females during the breeding season from the end of October to February (Shen and Liu, 2007; Qi et al., 2011; Hawkins, 1950a). However, immature musk is white and not intensely scented, as two phases are required for musk maturation (Wu and Wang, 2006). In the first phase, the initial musk liquid is secreted by the musk gland and enters the musk pod near the prepuce at the end of May. The second phase began at the end of June and finished at the end of October, during which the musk was maturing in the musk pod (Jie et al., 2014). Determining the path from immature to mature musk, the production of intense perfume, and what impacts the odor of musk has been inconclusive.

Existing studies have uncovered that natural musk is an excellent source of TCM pharmaceutical preparations, including bioactive components like muscone (Wang et al., 2020). The high demand for musk in TCM and perfumery drives unsustainable harvesting, making chemical characterization of musk essential for conservation efforts. Understanding the composition of musk can inform the development of synthetic substitutes, reducing reliance on wild populations. With improved analytical methods, metabolite measurement is more accessible and widespread (Wang et al., 2021a; Jin et al., 2013). According to the Chinese Pharmacopoeia (2020 edition), the concentration of muscone was determined by GC-MS and HPLC (Wang et al., 2021a) to control the quality of natural musk. The chemical composition of TCM is complicated, it is no longer suitable to rely solely on muscone as an index of biological activity with the appearance of synthetic muscone. As another kind of characteristic active substance in musk, the content of steroid is also a major characteristic to identify the authenticity of musk (Zhang et al., 2002). Zhang et al. (2002) used GC-MS spectroscopy and searched the NIST standard library to quickly determine most of the chemical composition in the musk sample, making it easier to screen for false musk. The chemical analysis can aid in detecting counterfeit or illegally sourced musk supporting anti-poaching initiatives and international trade regulations (e.g., CITES) (Ding et al., 2022). The characteristics of musk were determined by the distribution of key active ingredients in quality and quantity, including macrocyclic ketones, steroids, and polypeptides (Liu et al., 2021; Lv et al., 2022). Among the top twenty chemicals, ketones and alkanes were more represented in August and October, while the relative abundance of esters, acids, and alcohols decreased with maturity (Jie et al., 2021). High-quality musk is red-brown, and the concentration of muscone should exceed 2% according to GC-MS detection, with more muscone detected in unmated males (Zheng et al., 2020; Li et al., 2016). This suggests that the chemical composition of musk is complex and variable according to the musk secretion process, mated status, and age (Zheng et al., 2020; Xu et al., 2024; Yuan et al., 2021). However, the effects of musk maturation must be investigated across a wider spectrum to determine the dynamic alterations of the metabolite composition in musk.

In this study, we applied GC-MS to detect the chemical components of natural musk, the composition and classification of musk were categorized unambiguously. Our work uniquely combines “metabolomic profiling” with “biosynthetic pathway analysis” to identify potential precursors and enzymes involved in musk biosynthesis. This fills a gap in understanding how musk deer produce these compounds endogenously. The finding could accelerate the development of synthesized musk compounds sustainably, reducing reliance on wild populations.

## Material and methods

### Experimental animals

Six healthy 3-year-old male forest musk deer, weighing approximately 8.0 kg were fed separately in a 30 m^2^ enclosure at Chongqing Institute of Medicinal Plant Cultivation (altitude: 678 m).

### Dietary standardization and quality control

All musk deer received a strictly standardized diet as described follows. The concentrate-to-forage ratio (1:4) and ingredient percentages (e.g., 65% corn, 25% soybean, 6% wheat bran, 0.4% NaCl, 1.5% CaCO_3_, 0.8% CaHPO_4_, 0.15% multi-vitamin, and 0.15% muti-mineral) were uniformly applied to all individuals. Concentrate feed was purchased from Tongwei Group, China. Coarse fodders (e.g., *Ipomoea batatas*, *Pittosporum* leaves, *Broussonetia papyrifera* leaves, and other light green weeds) were harvested from the same geographical area and processed identically (e.g., air-dried, chopped to 2–3 cm lengths) to minimize compositional variation. Feeding times (08:30 and 17:30) and *ad libitum* water access were consistent across all animals to ensure dietary rhythmicity. Concentrate ingredients (corn, soybean) were sourced from a single supplier and analyzed for macronutrient content (e.g., crude protein, fiber) to confirm batch-to-batch consistency. Coarse fodder samples were periodically tested for moisture and secondary metabolites to ensure no seasonal or regional variations affected the diet during the study period.

### Collection of musk

**FIGURE 1:**
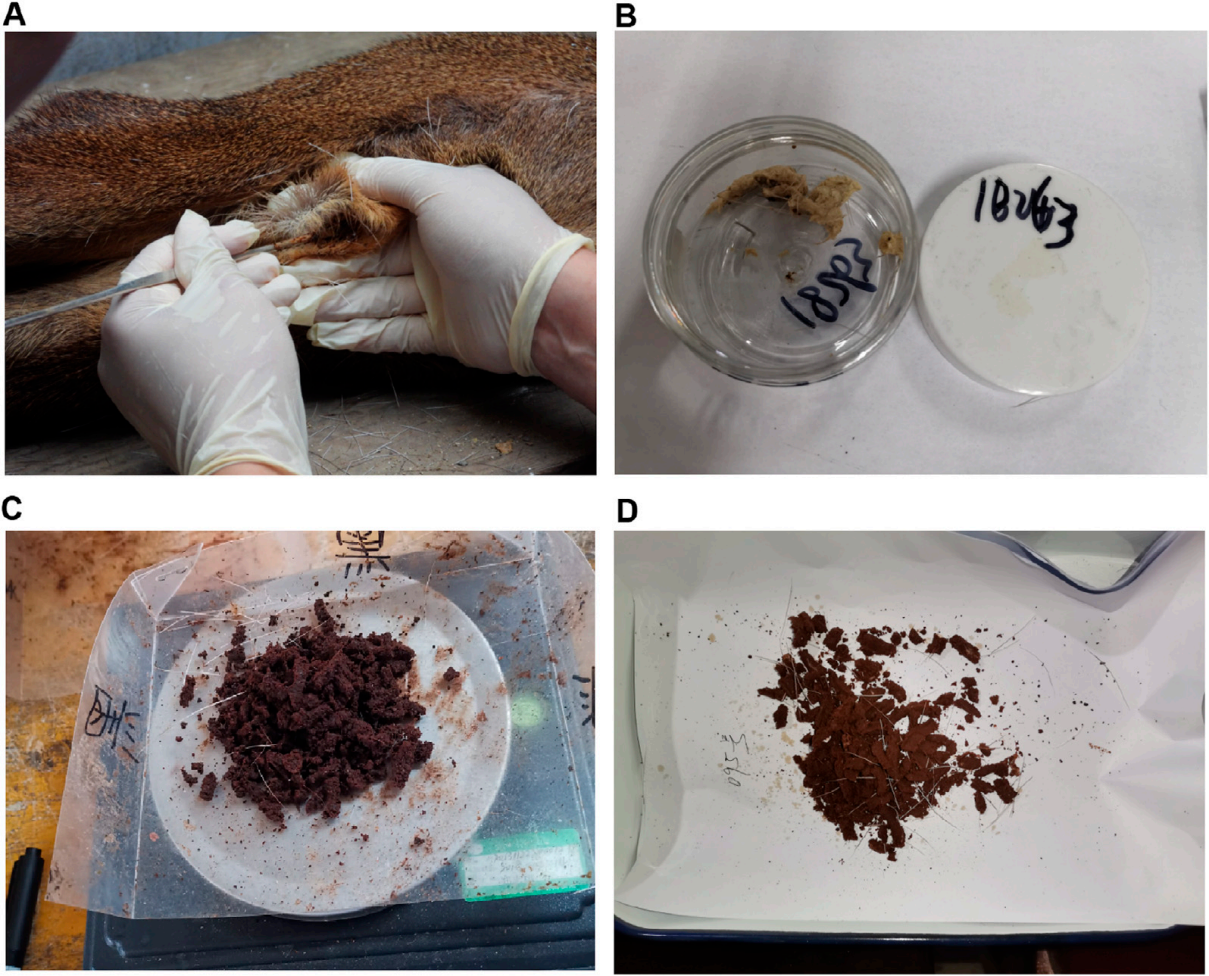
Musk collected at different maturity stages. **(A)** Schematic of musk collection approach. **(B)** The musk harvested at the end of June, in the initial stage. **(C)** The musk harvested at the end of August, during the maturing stage. **(D)** The musk harvested at the end of October, during the mature stage.

Initial mature musk was harvested on a cool morning at the end of June 2023. The selected forest musk deer was caught adhering to animal welfare guidelines. The skin surrounding the musk pod was disinfected using alcohol wipes. A special disinfected long-handle spoon was inserted into the musk scent pod and removed slowly, and this operation has no harm to forest musk deer itself, and will not affect musk secretion in the next year (Figure 1A). The forest musk deer was treated carefully under ethical consideration during sampling. Obtaining maturing and mature musk was the same as in the initial stage, but the collection time was the end of August and October 2023, respectively. A total of 18 musk samples (6 deer × 3 stages) were obtained. The minimum weight of each sample was 2.0 g, and each musk sample was placed in sterile cryogenic vials, flash-frozen in liquid nitrogen, and stored at −80°C until further experiments.

### Untargeted GC-MS metabolomics determination

Samples (∼60 mg, n = 18) were obtained in 2-mL EP tubes, and underwent extraction with 0.48 mL of extraction liquid (V methanol: V chloroform = 3:1). Samples were homogenized in a ball mill for 4 min at 45 Hz, then ultrasound treated for 5 min in ice water. The resulting homogenate was centrifuged for 15 min at 13,000 rpm, at 4°C, and the supernatant (0.4 mL) was transferred to a fresh 2-mL GC-MS glass vial. GC-MS analysis was performed using an Agilent 7890 gas chromatograph system (Agilent, United States) coupled to a Pegasus HT time-of-flight mass spectrometer (Leco, United States). The system employed a DB-5MS capillary column coated with 5% diphenyl cross-linked with 95% dimethylpolysiloxane (30 m × 250 μm inner diameter, 0.25 μm film thickness; J&W Scientific, Folsom, CA, United States). A 1-μL aliquot of the analyte was injected in splitless mode. Helium was utilized as the carrier gas, the front inlet purge flow was 3 mL min^−1^, and the gas flow rate through the column was 1 mL min^−1^. The initial temperature was maintained at 50°C for 1 min, then raised to 310°C at a rate of 10°C min^−1^, and held for 8 min at 310°C. The injection, transfer line, and ion source temperatures were 280, 270, and 220°C, respectively. The energy was −70 eV in electron impact mode. Mass spectrometry data were acquired using full-scan mode with a m/z range of 40–500 at a rate of 20 spectra per second following a solvent delay of 455 s.

### Metabolites identification

Chroma TOF 4.3X software from LECO Corporation and the NIST database were employed for raw peak extraction, data baseline filtering and calibration, peak alignment, deconvolution analysis, peak identification, and peak area integration. Peak intensities were normalized to the total spectral intensity and used to predict the molecular formula according to additive ions, molecular ion peaks, and fragment ions. Peaks were matched using the mzCloud, mzVault, and MassList databases to obtain accurate qualitative and relative quantitative results.

### Data analysis

To address technical variability in GC-MS data, all metabolite peak areas were scaled by the Total Ion Current (TIC) of each sample to correct for differences in injection volume and ionization efficiency (Wishart, 2016). Normalized data were log2-transformed to stabilize variance and approximate normality, improving the robustness of parametric statistical tests. We used the IQR method to identify and filter outliers in the normalized data: For each metabolite, calculate the first quartile (Q1) and third quartile (Q3) across all samples, define the IQR as Q3-Q1, flag values outside the range [Q1 − 1.5 × IQR, Q3 + 1.5 × IQR] as outliers, finally to remove these outliers (van den Berg, 2006). We additionally filtered metabolites with ≥50% missing values in peak area across all groups (Broadhurst et al., 2018). A total of 80 normalized data was obtained from preprocessed raw data. Metabolites were mapped to PubChem, HMDB, and KEGG databases to determine annotation and classification information for subsequent analysis. Multivariate statistical analysis, including principal component analysis (PCA) and hierarchical cluster analysis (HCA), was conducted using R packages. The processed data were log-transformed and mean-centered to perform orthogonal partial least squares discriminate analysis (OPLS-DA), from which the VIP (Variable Importance in the Projection) value was determined. The trend analysis was conducted by K-means, and chemicals with VIP > 1 and *P* < 0.05 were differential metabolites for the two groups. The enrichment and pathway analysis were performed to develop a network using the KEGG database.

## Results and discussion

### Properties of musk obtained at different maturities

The musk gland synthesizes and secretes musk, which enters the well-developed musk pod for storage, where it develops for about 2 months before reaching maturity (Jiang et al., 2023; Chen et al., 2018; Liu et al., 2023). Premature and final musk isolated from musk pods were normal, while their physical properties across maturity stages varied (Li et al., 2018; Zhang et al., 2021). Secretions obtained at the end of June were white and wet, like soybean curb residue (Figure 1B), and had strong, foul odors. In contrast, secretions at the end of August were light brown, oily semi-solid, like oily earwax (Figure 1C), and had a refreshing and enduring fragrance. The mature musk collected at the end of October was deep brown, oily powder or granules, like dry earwax (Figure 1D), with refreshing, enduring, and fascinating scents. Browning was involved in the transition from the milky initial liquid to the brown solid, with mature musk containing volatile aromatic intermediates like aldehydes, ketones, pyridines, and furans. Musk is considered a pheromone for marking territories and attracting females, enabling discrimination of strong individuals (Jiang et al., 2023; Hawkins, 1950b). Normal secretions collected at the final stage were high-quality black-brown solid musk (Wang et al., 2022), characterized by high levels of muscone and low levels of moisture (Zheng et al., 2020; Fan et al., 2018). The relative abundance of muscone was stable, while cholesterols and steroids changed in natural musk (Su et al., 2009).

### Overview of the metabolites identified in musk

The chemical composition was related to the quality, potentially indicating the metabolic profile of natural musk (Wang et al., 2022; Wang et al., 2021b). We utilized untargeted GC-MS metabolomics to identify 93 chemical compounds, encompassing 80 known and 13 unidentified metabolites, comparable to prior studies (Lv et al., 2022). The remaining 80 chemicals, consisting of 66 known and 14 unknown chemicals, were used for subsequent analysis. Based on chemical classification, the 66 known metabolites were categorized into five subclasses, and 29 chemicals were assigned as aromatic compounds (Supplementary Table S1).

**FIGURE 2:**
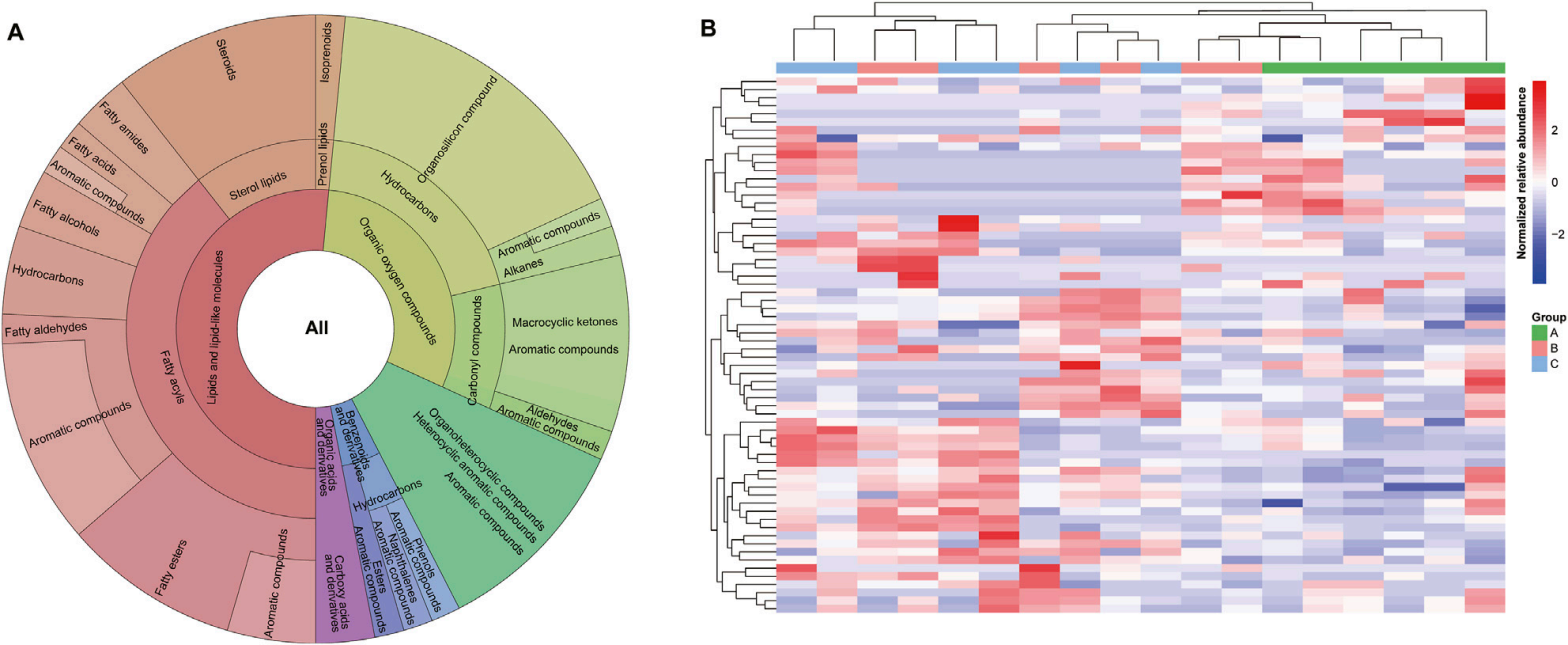
Metabolite analysis of musk across three stages. **(A)** Classification of known metabolites. The hierarchical pie chart includes 66 metabolites, superclass, class, subclass, and aromatic compounds from the inside out, respectively. **(B)** HCA results of all metabolites in all samples. A, B, and C represent metabolites from June, August, and October, respectively.

Lipids are primary odor carriers and potential precursors of odorous substances (Sokolov et al., 1987). The Krona chart demonstrated that we isolated 34 (51.52%) lipids and lipid-like molecules, the highest proportion among metabolites, including 26 fatty acyls (FA), seven sterol lipids (ST), and one prenol lipid (PL). Among them, 11 chemicals belonging to FA (seven aldehydes [FA06], two esters [FA07], and one acid [FA01]) were aromatic compounds (Figure 2A; Supplementary Table S1). Three chemicals, namely, (Z)-13-Octadecenal, 9-Octadecenal, and cis-11-Hexadecenal (aromatic compounds), as well as 6-methyl-5-Hepten-2-ol, were active ingredients (AI) in the Traditional Chinese Medicine Systems Pharmacology Database and Analysis Platform (TCMSP) (Ru et al., 2014). Additionally, there were six C19 steroids (androgens) and derivatives [ST0202] and one cholesterol and derivatives [ST0101], the most common component in natural musk (Sokolov et al., 1987; Xie et al., 2022). These compounds had crucial functions in the chemical communication exchange between muskrats by regulating pheromone synthesis in musk cells (Jiang et al., 2022). Which illustrates a more systematic and comprehensive classification than previous studies (Liu et al., 2021; Lv et al., 2022; Zhang et al., 2021). Such as more detailed classification of lipids including fatty acyls (FA: FA01, FA06, FA07), sterol lipids (ST), and prenol lipid (PL). The AIs and targets of musk were studied from the perspective of systematic pharmacology through TCMSP database.

Organic oxygen compounds were the second most abundant compounds in musk (28.79%), encompassing 13 hydrocarbons and seven carbonyl compounds. 3-Methylcyclopentadecanone (muscone), which has not been identified in the secretion lipids (Sokolov et al., 1987), was isolated from the latter fraction that consisted of six macrocyclic ketones and one aldehyde (Figure 2A; Supplementary Table S1). Muscone, as the most abundant macrocyclic ketone, is a sweet, animal, and fatty compound (Shmuel, 2004). Together with low levels of analogs, it was the main medicinal active and odor-contributing ingredient in natural musk. Alkanes of organosilicon compounds, like tetradecyl oxirane, were also biologically active and aromatic compounds targeted to sodium-dependent noradrenaline transporters and nuclear receptor coactivator 2 associated with depression (Ru et al., 2014).

The third most abundant metabolites in musk were organoheterocyclic compounds (12.12%), and all were heterocyclic aromatic ingredients derived from furan, pyran, and pyridine compounds (Figure 2A; Supplementary Table S1) forming five- and six-membered heterocyclic rings (Majumdar et al., 2012). The aromaticity of furans is due to one of the lone pairs of electrons on the oxygen atom being delocalized into the ring, creating a 4n+2 aromatic system akin to benzene, which is formed by the oxidation of polyunsaturated fatty acids and carotenoids (Seok et al., 2015; Mariotti et al., 2013).

Additionally, three benzenoids and derivatives (4.54%, odoriferous constituents), and two organic acids and derivatives (3.03%) were detected in musk. Benzeneacetic acid, methyl ester, and p-Cresol were active ingredients (Figure 2A; Supplementary Table S1). Methyl phenylacetate (known as benzeneacetic acid, methyl ester) is related to various diseases such as cardiovascular disease, neurogenic disease, inflammation, thromboembolic disorders, and cancer (Ru et al., 2014). p-Cresol was related to alcoholism and leukemia by targeting alcohol dehydrogenases and nicotinate-nucleotide-dimethylbenzimidazole phosphoribosyltransferase (Ru et al., 2014).

By performing multivariate analyses on samples (Hockenhull et al., 2012), we can characterize the overall metabolite differences and the degree of variability between samples throughout musk maturation. The heatmap of HCA among all normalized data after quality control demonstrated that the metabolic spectrum of initial musk liquid in June was distinct from those of August and October (Figure 2B).

### Identification of differential metabolites during musk maturation

**FIGURE 3:**
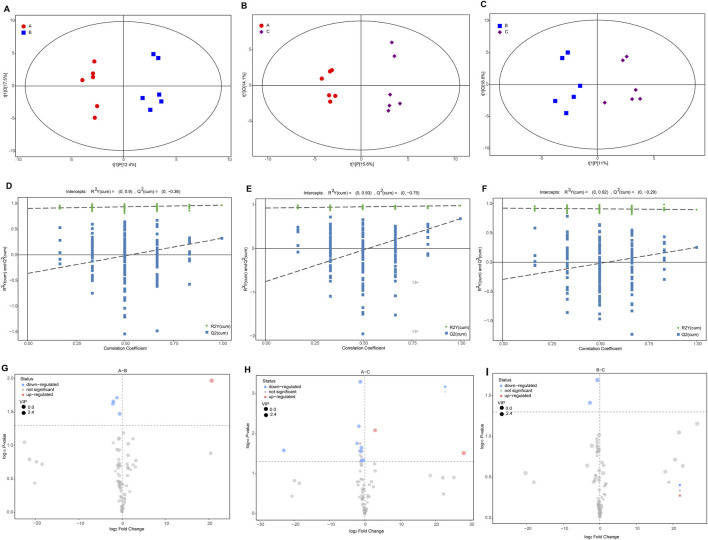
Multivariate and differential analyses of metabolites in musk. **(A–C)** Partial least squares discriminant analysis (PLS-DA) between different groups. **(D–F)** PLS-DA models were validated by permutation tests (n = 6). **(G–I)** Volcano plots depict the number of differential metabolites between groups.

The OPLS model, integrating multivariate statistical data with supervised pattern recognition, successfully distinguished two groups (Figures 3A,B,C), illustrating significant separation in metabolite levels between different groups. Permutation tests illustrated the R^2^ data were higher than Q^2^, R^2^Y explained 0.968 (A vs. B), 0.984 (A vs. C), and 0.901(B vs. C) goodness of fit of this model, respectively. And Q^2^ explained 0.323 (A vs. B), 0.254(A vs. C), 0.439(B vs. C) goodness of prediction, respectively. And all figures show high R^2^Y (cum) values (0.90–0.93), indicating strong data fit (Figures 3D,E,F).

The distribution of identified significantly differential metabolites in musk during maturation was illustrated by volcano plots following VIP > 1, *P* < 0.05 (Figure 3G). Among these, one was significantly up- and four were downregulated metabolites in the comparison between June and August. Ten of the 80 metabolites (12.50%), including two up- and eight downregulated chemicals, were significantly different when comparing June and October (Figure 3H). Two chemicals experienced a downward trend when comparing August and October (Figure 3I). Nine (out of 13) lipids and lipid-like molecules were significantly different between groups, including four aromatic compounds, namely, Methyl tetradecanoate, Tetracosanoic acid, methyl ester, cyclopentadecanone, and cis-11-Hexadecenal. This suggests that lipids are essential during musk secretion and maturation, in addition to one benzenoid and derivatives (p-Cresol, aromatic compounds), one organoheterocyclic compound (2-butyltetrahydrofuran, aromatic compounds), and an organosilicon compound (Supplementary Table S2).

### Metabolite trend analysis during musk maturation

**FIGURE 4:**
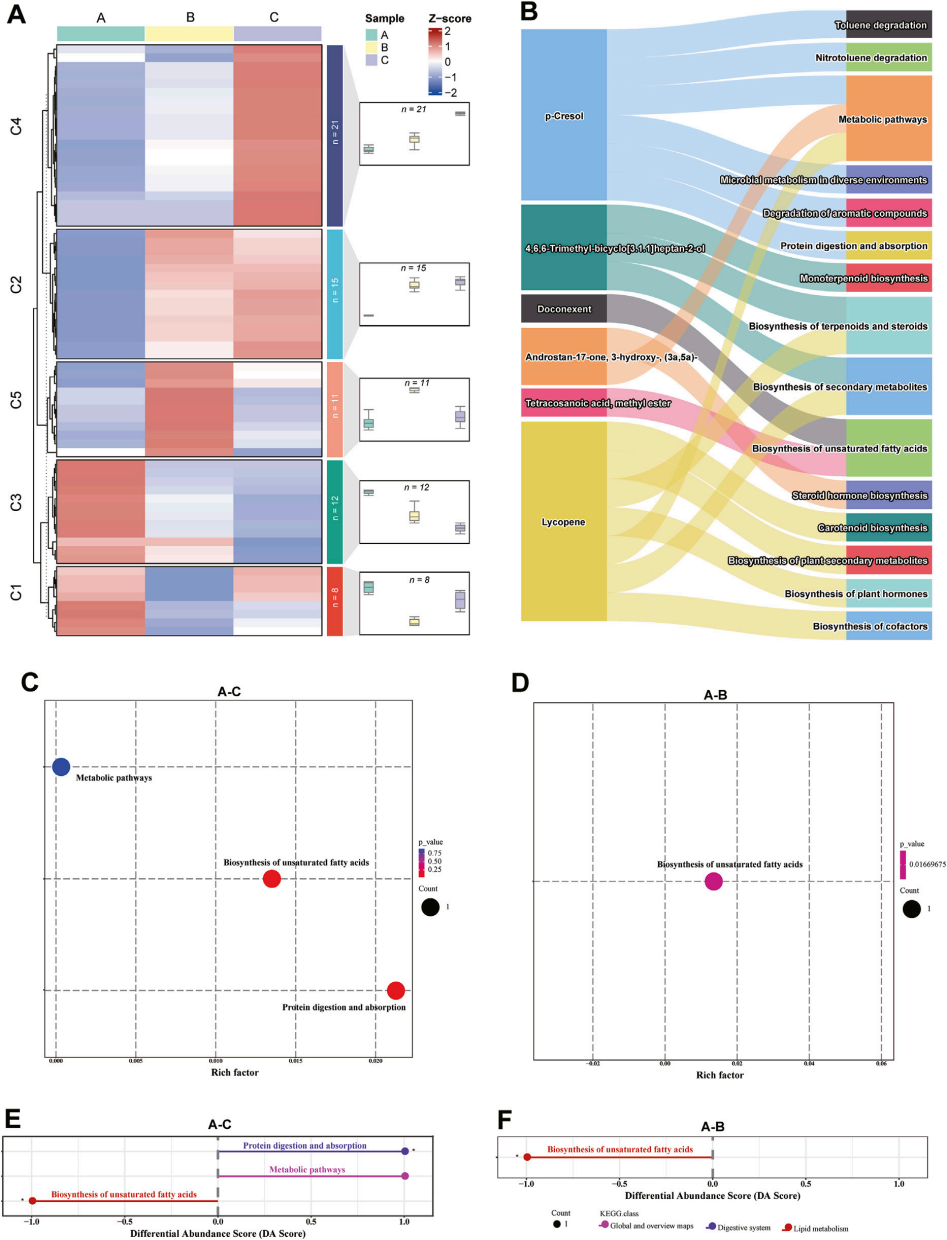
Dynamic profiles and enriched pathways of metabolites during musk maturation. **(A)** Metabolites were enriched into six clusters by K-means analysis. The number indicates the number of metabolites in the corresponding cluster. **(B)** Annotated metabolites and their associated pathways. **(C,D)** KEGG enrichment analysis of differential metabolites between groups. **(E,F)** Differential abundance (DA) score of metabolic pathways between groups.

The relative abundance of the identified 80 metabolites was standardized using a z-score, identifying 67 chemicals assigned to 5 groups throughout musk maturation by conducting trend analysis (Drakopoulos et al., 2016). A total of 36 metabolites were enriched into increased trends (C2 and C4), 12 chemicals (C3) exhibited downward trends, and the relative abundance of metabolites in C1 (V-shaped, n = 8) and C5 (Bell-shaped, n = 11) were assigned to opposing trends (Figure 4A; Supplementary Table S3). As heterocyclic aromatic compounds (chemical 4, 5, 8, 10, and 25), fatty acids (chemicals 7 and 77), phenols (chemical 16), and hydrocarbon (chemical 47) were gradually consumed, the relative concentration of four macrocyclic ketones containing C15 (chemical 49) and C16 (chemicals 44, 51 and 53) in cluster 4, as well as the six androgens and derivatives (chemicals 74, 76, 79, 83, 87, and 93) was increased. Macrocyclic ketones [(Cyclopentadecanone (C15) and 5α-androstan-3β-ol-17-one acetate (C16)] in C4 are key components of musk, they are structurally similar to muscone (a C16 macrocyclic ketone), the signature compound in natural musk (Liu et al., 2021). Macrocyclic ketones provide the characteristic musky aroma critical for communication (e.g., attracting mates or marking territories) (Li et al., 2016). Their accumulation likely reflects enzymatic oxidation of fatty acids/hydrocarbons (e.g., chemical 47) via cytochrome P450 pathways, a process conserved in musk biosynthesis across species (Han et al., 2017). Testosterone and its metabolites regulate musk gland development and secretion timing (Xie et al., 2020). Androgens may act as precursors for odoriferous compounds or enhance scent persistence via interactions with macrocyclic ketones (Liu et al., 2019). Consumption of heterocyclic aromatics (chemicals 4, 5) and fatty acids (chemical 7) likely diverts resources toward macrocyclic ketone biosynthesis, especially cyclopentadecanone and 5 alpha-androstan-3 beta-ol-17-one acetate, were significantly different metabolites during maturation. Hinting a coordinated biochemical network where macrocyclic ketones (C4) serve as the aromatic core, while androgens and derivatives modulate their biosynthesis and biological activity. Intriguingly, eight and two of the 13 significantly differential metabolites increased and decreased, respectively. An overwhelming majority (seven chemicals) of the increased class were lipids and lipid-like molecules. As presented in Supplementary Figure S1, the Venn diagram presented the number of metabolites in each comparison group, and the overlap between metabolites among different groups. 5,8,11-Eicosatriynoic acid, methyl ester was varied over three stages, and the unique number of metabolites in the A-C and A-B groups were 7 and 3, respectively.

#### Metabolite annotation

Due to the complexity and variability of chemical composition in natural musk and the fact that most of the compounds are derivatives, limited metabolite mapping information was obtained on the KEGG database. A total of six metabolites were successfully annotated in the KEGG Compound database, and 15 pathways were assigned to the KEGG Pathway database (Figure 4B; Supplementary Table S4). Among the ten differentially expressed metabolites between June and October, three pathways (biosynthesis of unsaturated fatty acids, metabolic pathway, and protein digestion and absorption) were enriched in p-Cresol and tetracosanoic acid, methyl ester. Biosynthesis of unsaturated fatty acids was assigned to tetracosanoic acid, methyl ester in the June and August comparison group. There was no pathway enrichment in the chemicals of the August and October comparison group (Figures 4C,D). The differential abundance (DA) score of metabolic pathways between the comparison groups was illustrated in Figures 4E,F. This demonstrates the enriched pathway “biosynthesis of unsaturated fatty acids” was upregulated while “metabolic pathway” and “protein digestion and absorption” were downregulated in June.

#### Pathways involved in the synthesis of key bioactive metabolites

Various active ingredients, including macrocyclic ketones, steroids, polypeptides, and proteins, have been isolated from natural musk (Liu et al., 2021; Lv et al., 2022). Muscone (3-methylcyclopentadecanone) is the main active ingredient, whose biosynthesis remains unclear, although its initial discovery was in 1906 (Fujimoto et al., 2002). Cyclopentadecanone is a chemosynthetic precursor of muscone (McGintyc et al., 2011). Macrocyclic musk compounds such as muscone, cyclopentadecanone, 5-Cyclohexadecen-1-one, 8-Cyclohexadecen-1-one, and 9,19-Cyclolanostan-24-one, 3-acetoxy-25-methoxy- were detected in our study, known as cyclic ketones, containing a ketone conjugated to a cyclic moiety (Shmuel, 2004).

**FIGURE 5:**
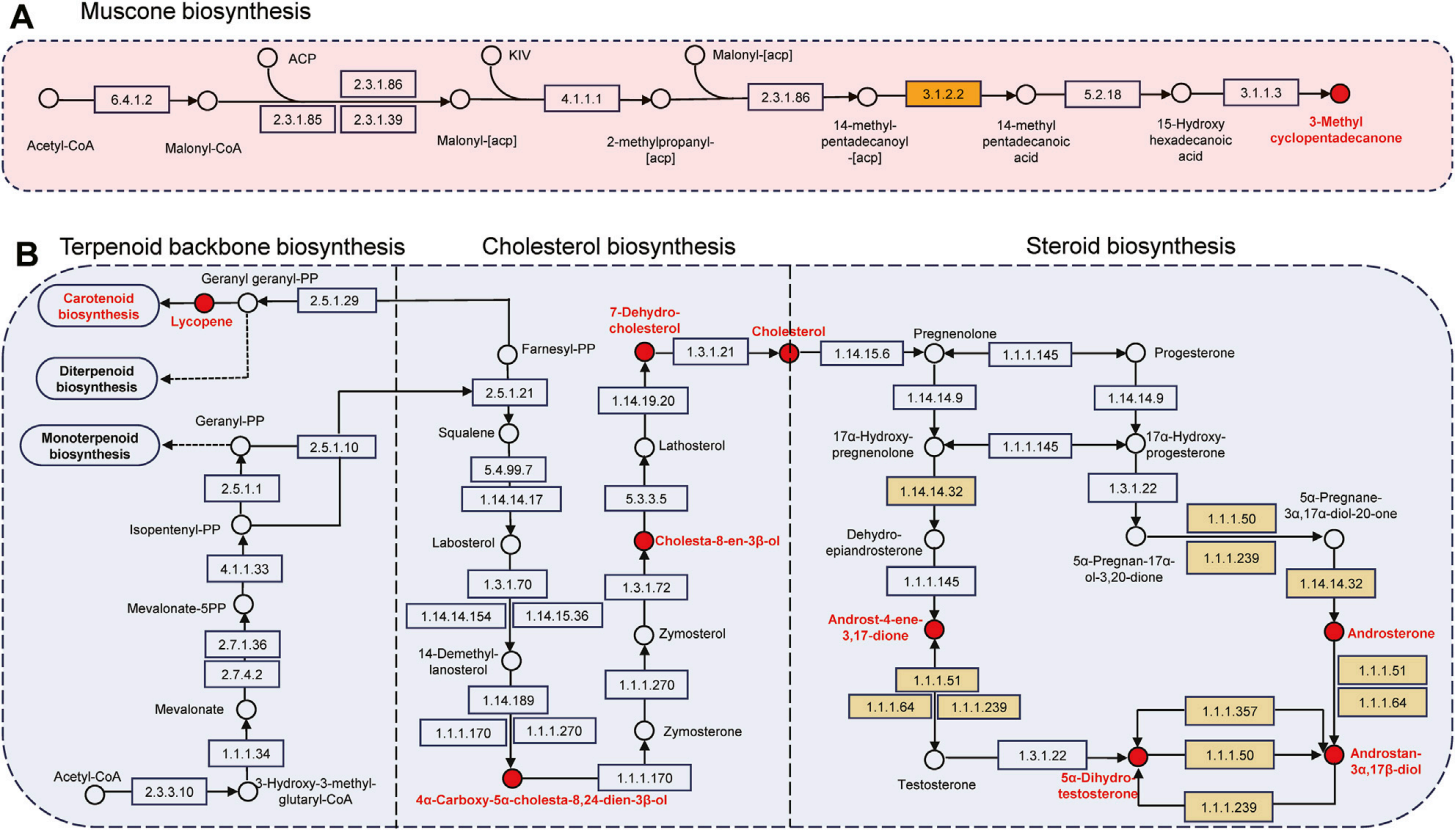
Pathways involved in bioactive chemical synthesis. **(A)** Muscone biosynthesis. **(B)** Steroid biosynthesis. “○” represents chemical compounds, and “□” indicates enzymes. Red chemicals and yellow enzymes were identified in this paper.

The pathways and associated chemicals had not been previously investigated due to the complexity of macrocyclic musk biosynthesis. Acetyl-CoA is a metabolite derived from glucose, fatty acid, and amino acid through the Krebs cycle (Metallo et al., 2011). As illustrated in Figure 5A, its production is catalyzed by acetyl-CoA carboxylase (ACC1, [EC: 6.4.1.2]) to produce malonyl-CoA, together with ACP and fatty acid synthases (FASN [EC: 2.3.1.85], FAS1 [EC: 2.3.1.86] and Fas [EC: 2.3.1.39]) to form malonyl-[acp]. This synthetic precursor was catalyzed by several enzymes, including pyruvate decarboxylase (THI3, [EC: 4.1.1.1]), fatty acid synthase subunit (FAS1 and 2, [EC: 2.3.1.86]), and palmitoyl-CoA hydrolase (TES1, [EC: 3.1.2.2]) to participate in fatty acid elongation and synthesis of 14-methylpentadecanoic acid. The product was then hydroxylated by cytochrome P450 monooxygenase 54 and cytochrome P450 cyp2 (CYP54 and CYP2, [EC: 5.2.1.8]) to produce 15-Hydroxyhexadecanoic acid, and cyclized by lactonizing lipase (LipL, [EC: 3.1.1.3]) to produce muscone. Palmitoyl-CoA hydrolase [EC: 3.1.2.2]), which hydrolyzes CoA thioesters of long-chain fatty acids, participates in the biosynthesis of unsaturated fatty acids like doconexent and tetracosanoic acid, methyl ester in our biosynthesis (Figure 5A; Supplementary Table S5). Musk resources have been considerably reduced due to overhunting, leading to research hindrances paving the way for synthetic musk production (Wang et al., 2023). Numerous chemicals, including citronellal, citronellic acid, pentadecanedioic acid, tetradecanedioic acid, methyl undecenate, and 1,10-dibromodecane are chemosynthetic precursors of macrocyclic musk (Lv et al., 2022; Fujimoto et al., 2002; Latscha and Kazmaier, 2016). In this study, we speculated that lipids, including fatty acids, aldehydes, and esters, were potential precursors in macrocyclic musk biosynthesis.

Terpenoids, cholesterols, and steroid hormones were closely related to the steroid biosynthetic pathway (Figure 5B; Supplementary Table S5). For instance, lycopene is a terpene assembled from 8 isoprene units and a bright red carotenoid pigment, it is one of the most potent carotenoid antioxidants and exhibits chemopreventive properties (Yeh and Hu, 2000). The red-brown presented in mature musk probably related to the carotenoid pigmentation. Cholesterol is critical in lipid metabolism and operates in signal transduction and sperm development (Lingwood and Simons, 2010). It is a biosynthetic precursor of steroid hormones (androstenone and derivatives) and contributes to central nervous system functioning (Simons and Toomre, 2000). The genetic mechanisms of steroidogenesis have been more comprehensively explored in the scent glands of Chinese forest deer and muskrats (Wang et al., 2025; Jiang et al., 2022; Feng et al., 2023). We identified seven androgens and derivatives (e.g., chemical 74, 76, 79, 83, 86, 87, and 93), which function as pheromones and mammalian metabolites. A total of six steroidogenic enzymes, including CYP17A1 (EC: 1.14.14.32), AKR1C (EC: 1.1.1.51), HSD17B3 (EC: 1.1.1.64), HSD17B (EC: 1.1.1.239), AKR1C4 (EC: 1.1.1.50), and AKR1C2 (EC: 1.1.1.357) participate in steroid hormone biosynthesis. During this time, CYP17A1 was one of the three key rate-limiting enzymes; it was localized in the musk glandular ducts of forest musk deer and involved in the local synthesis of androgens (Feng et al., 2023).

## Conclusion

To date, studies on the composition of natural musk have primarily focused on characterizing contents and their quantities. However, relatively few comprehensive analyses of the pathways involved in the biosynthesis of metabolites in natural musk, have been undertaken. We have mapped the metabolites to multiple databases and defined them systematically. Macrocyclic ketones were determined as organic oxygen compounds, and lipids were categorized unambiguously. Additionally, a deciphered biosynthetic pathway of muscone and steroids was constructed according to the compound-pathway-enzyme-reaction network analysis. This deepens the understanding of natural musk composition and offers new theoretical insights to develop and utilize musk, such as engineering bacteria modification to establish a high-efficiency biosynthesis system for muscone in a targeted manner (Zhang, 2022), and thereby reducing the demand for wild forest musk deer resources. While our metabolomic data suggest involvement of muscone/steroid biosynthesis pathways, experimental validation (e.g., enzyme activity assays, isotope tracing) is required to confirm these mechanistic links. Future studies should integrate multi-omics approaches (metabolomics + transcriptomics + proteomics) to dissect the genetic and enzymatic basis of musk maturation.

## Data Availability

The metabolomics data (GC-MS) of 18 samples from *Moschus berezovskii* (forest musk deer) were deposited in the China National Center for Bioinformation (CNCB) under the accession number OMIX009672. Available at: https://ngdc.cncb.ac.cn/omix/releaseList.

## Abbreviations

GC-MS, Gas Chromatography-Mass Spectrometry; EC, Enzyme Commission; IUCN, International Union for Conservation of Nature; TCM, Traditional Chinese Medicine; HMDB, The Human Metabolome Database; KEGG, The Kyoto Encyclopedia of Genes and Genomes; PCA, Principal Component Analysis; HCA, Hierarchical Cluster Analysis; OPLS-DA, Orthogonal Partial Least Squares Discriminate Analysis; VIP, Variable Importance in the Projection; FA, Fatty Acyls; ST, Sterol Lipids; PL, Prenol Lipid; AI, Active Ingredients; TCMSP, Traditional Chinese Medicine Systems Pharmacology Database and Analysis Platform; DA, Differential Abundance.

## References

[B1] Broadhurst D. Goodacre R. Reinke S. N. Kuligowski J. Wilson I. D. Lewis M. R. (2018). Guidelines and considerations for the use of system suitability and quality control samples in mass spectrometry assays applied in untargeted clinical metabolomic studies. Metabolomics 14, 72. 10.1007/s11306-018-1367-3 29805336 PMC5960010

[B2] Chen M. Jie H. Xu Z. Ma T. Lei M. Zeng D. (2018). Isolation, primary culture, and morphological characterization of gland epithelium from forest musk deer (*Moschus berezovskii*). Vitro Cell Dev. Biol. Anim. 54, 545–548. 10.1007/s11626-018-0268-0 30083840

[B3] Ding M. Fan J.-L. Huang D.-F. Jiang Y. Li M.-N. Zheng Y.-Q. (2022). From non-targeted to targeted GC-MS metabolomics strategy for identification of TCM preparations containing natural and artificial musk. Chin. Med. 17, 41. 10.1186/s13020-022-00594-8 35365201 PMC8974109

[B4] Drakopoulos G. Gourgaris P. Kanavos A. Makris C. (2016). A fuzzy graph framework for initializing k-means. Int. J. Artif. Intell. Tools 25, 1650031. 10.1142/s0218213016500317

[B5] Ehlers S. Schulz S. (2023). The scent chemistry of butterflies. Nat. Prod. Rep. 40, 794–818. 10.1039/d2np00067a 36420976

[B6] Fan M. Zhang M. Shi M. Zhang T. Qi L. Yu J. (2018). Sex hormones play roles in determining musk composition during the early stages of musk secretion by musk deer (*Moschus berezovskii*). Endocr. J. 65, 1111–1120. 10.1507/endocrj.EJ18-0211 30175720

[B7] Feng H. Feng T. Mo Y. Sun S. Wang L. Lu C. (2023). Integrated multi-omics analysis reveals insights into Chinese forest musk deer (*Moschus berezovskii*) genome evolution and musk synthesis. Front. Cell Dev. Biol. 11, 1156138. 10.3389/fcell.2023.1156138 37228656 PMC10203155

[B8] Fujimoto S. Yoshikawa K. Itoh M. Kitahara T. (2002). Synthesis of (R)- and (S)- muscone. Biochemistry 66, 1389–1392. 10.1271/bbb.66.1389 12162565

[B9] Geng S. Ma S. (2000). Decline of musk deer in China and prospects for management. Environ. Conserv. 27, 323–325. 10.1017/s0376892900000369

[B10] Han W. Xie W. Zhang Y. Zhang F. Zhang H. Han Y. (2017). Seasonal expression of P450c17 and 5α-reductase-2 in the scented gland of male muskrats (*Ondatra zibethicus*). Gen. Comp. Endocrinol. 254, 60–67. 10.1016/j.ygcen.2017.09.015 28919450

[B12] Hawkins T. H. (1950a). Musk and the musk deer. Nature 166, 262. 10.1038/166262a0 15439270

[B13] Hawkins T. H. (1950b). Musk and the musk deer. Nature 166, 262. 10.1038/166262a0 15439270

[B14] Hockenhull J. C. Whittington R. Leitner M. Barr W. Mcguire J. Cherry M. G. (2012). A systematic review of prevention and intervention strategies for populations at high risk of engaging in violent behaviour: update 2002-8. Health Technol. Asses 16, 1–152. 10.3310/hta16030 PMC4781592 22330980

[B15] Jiang Y. Han X. Feng N. Jin W. Zhang T. Zhang M. (2022). Androgen plays an important role in regulating the synthesis of pheromone in the scent gland of muskrat. J. Steroid Biochem. Mol. Biol. 217, 106026. 10.1016/j.jsbmb.2021.106026 34808361

[B17] Jie H. Feng X. L. Zhao G. J. Zeng D. J. Chen Q. (2014). Research progress on musk secretion mechanism of forest musk deer. Zhongguo Zhong Yao Za Zhi 39, 4522–4525. 10.4268/cjcmm20142309 25911794

[B18] Jie H. Zhang C. L. Zeng D. J. Zhao G. J. Feng X. L. Lei M. (2021). Variation of chemical constituents in musk harvested at different maturity stages (in Chinese). Chin. Tradit. Pat. Medcine 43, 144–148. 10.3969/j.issn.1001-1528.2021.01.028

[B19] Jin C. Yan C. Luo Y. Li B. He J. Xiao X. (2013). Fast and direct quantification of underivatized muscone by ultra performance liquid chromatography coupled with evaporative light scattering detection. J. Sep. Sci. 36, 1762–1767. 10.1002/jssc.201200946 23520031

[B20] Latscha H. P. Kazmaier U. (2016). Aldehyde, ketone und chinone. Berlin, Heidelberg: Springer Spektrum.

[B21] Li D. Chen B. Zhang L. Gaur U. Ma T. Jie H. (2016). The musk chemical composition and microbiota of Chinese forest musk deer males. Sci. Rep. 6, 18975. 10.1038/srep18975 26744067 PMC4705530

[B22] Li Y. Zhang T. Qi L. Yang S. Xu S. Cha M. (2018). Microbiota changes in the musk gland of male forest musk deer during musk maturation. Front. Microbiol. 9, 3048. 10.3389/fmicb.2018.03048 30619139 PMC6297183

[B23] Lingwood D. Simons K. (2010). Lipid rafts as a membrane-organizing principle. Science 327, 46–50. 10.1126/science.1174621 20044567

[B24] Liu C. Hong T. Yu L. Chen Y. Wang S. Ren Z. (2023). Single-nucleus RNA and ATAC sequencing uncovers the molecular and cellular characteristics in the musk gland of Chinese forest musk deer (*Moschus berezovskii*). FASEB J. 37, e22742. 10.1096/fj.202201372R 36583723

[B25] Liu K. Xie L. Deng M. Zhang X. Luo J. Li X. (2021). Zoology, chemical composition, pharmacology, quality control and future perspective of Musk (*Moschus*): a review. Chin. Med. 16, 46. 10.1186/s13020-021-00457-8 34147113 PMC8214773

[B26] Liu Q. Yu W. Fan S. Zhuang H. Han Y. Zhang H. (2019). Seasonal expressions of androgen receptor, estrogen receptors, 5α-reductases and P450arom in the epididymis of the male muskrat (*Ondatra zibethicus*). J. Steroid Biochem. Mol. Biol. 194, 105433. 10.1016/j.jsbmb.2019.105433 31376460

[B27] Lv S. Lei Z. Yan G. Shah S. A. Ahmed S. Sun T. J. J. E. (2022). Chemical compositions and pharmacological activities of natural musk (*Moschus*) and artificial musk. A Rev. 284, 114799. 10.1016/j.jep.2021.114799 34748869

[B28] Majumdar K. C. Karunakar G. V. Sinha B. (2012). Formation of five- and six-membered heterocyclic rings under radical cyclization conditions. Synthesis Int. J. Methods Synthetic Org. Chem. 44, 2475–2505. 10.1055/s-0032-1316566

[B29] Mariotti M. S. Toledo C. Hevia K. Gomez J. P. Fromberg A. Granby K. (2013). Are Chileans exposed to dietary furan? Food Addit. Contam. Part A Chem. Anal. Control Expo. Risk Assess. 30, 1715–1721. 10.1080/19440049.2013.815807 23875686

[B30] McGintyc D. Letiziaa C. S. Api A. M. (2011). Fragrance material review on cyclopentadecanone. Food Chem. Toxicol. 49, S142–S148. 10.1016/j.fct.2011.07.041 21801797

[B31] Meng X. Liu D. Feng J. Meng Z. (2012). Asian medicine: exploitation of wildlife. Science 335, 1168. 10.1126/science.335.6073.1168-a 22403369

[B32] Metallo C. M. Gameiro P. A. Bell E. L. Mattaini K. R. Yang J. Hiller K. (2011). Reductive glutamine metabolism by IDH1 mediates lipogenesis under hypoxia. Nature 481, 380–384. 10.1038/nature10602 22101433 PMC3710581

[B33] Pharmacopoeia Commission (2020). Commission CP: Pharmacopoeia of the People's Republic of China. Beijing: China Medical Science and Technology Press.

[B34] Qi W. H. Li J. Zhang X. Y. Wang Z. K. Li X. X. Yang C. Z. (2011). The reproductive performance of female Forest musk deer (*Moschus berezovskii*) in captivity. Theriogenology 76, 874–881. 10.1016/j.theriogenology.2011.04.018 21664670

[B35] Ru J. Li P. Wang J. Zhou W. Li B. Huang C. (2014). TCMSP: a database of systems pharmacology for drug discovery from herbal medicines. J. Cheminform 6, 13. 10.1186/1758-2946-6-13 24735618 PMC4001360

[B36] Seok Y.-J. Her J.-Y. Kim Y.-G. Kim M. Y. Jeong S. Y. Kim M. K. (2015). Furan in thermally processed foods - a review. Toxicol. Res. 31, 241–253. 10.5487/TR.2015.31.3.241 26483883 PMC4609971

[B37] Shen H. Liu Z. (2007). The musk deer in China (in Chinese). Shanghai: Shanghai Scientific and Technical Publishers.

[B38] Shmuel Y. (2004). Dictionary of food compounds with CD-ROM: additives, flavors, and ingredients. Boca Raton: Chapman and Hall/CRC.

[B39] Simons K. Toomre D. (2000). Lipid rafts and signal transduction. Nat. Rev. Mol. Cell Biol. 1, 31–39. 10.1038/35036052 11413487

[B40] Sokolov V. E. Kagan M. Z. Vasilieva V. S. Prihodko V. I. Zinkevich E. P. (1987). Musk deer (*Moschus moschiferus*): reinvestigation of main lipid components from preputial gland secretion. J. Chem. Ecol. 13, 71–83. 10.1007/BF01020352 24301360

[B41] Su G. Y. Wu A. L. Gan X. N. Yue B. S. Li J. (2009). Quantitative analysis of musk components by gas chromatography/mass spectrometry (in Chinese). Sichuan J. Zoology 28, 2472–2482.

[B42] Thiessen D. Rice M. (1976). Mammalian scent gland marking and social behavior. Psychol. Bull. 83, 505–539. 10.1037/0033-2909.83.4.505 822437

[B43] van den Berg C. M. G. (2006). Chemical speciation of iron in seawater by cathodic stripping voltammetry with dihydroxynaphthalene. Anal. Chem. 78, 156–163. 10.1021/ac051441+ 16383323

[B44] Wang J. Xing H. Qin X. Ren Q. Yang J. Li L. (2020). Pharmacological effects and mechanisms of muscone. J. Ethnopharmacol. 262, 113120. 10.1016/j.jep.2020.113120 32668321

[B45] Wang S. Shang Y. Liang C. Liu T. Chang Y. X. Guo J. (2021b). Binary eluent based vortex-assisted matrix solid-phase dispersion for the extraction and determination of multicomponent from musk by gas chromatography-mass spectrometry. J. Anal. methods Chem. 2021, 9913055. 10.1155/2021/9913055 34422434 PMC8378966

[B46] Wang S. Shang Y. Liang C. Liu T. Du K. Guo J. (2021a). Binary eluent based vortex-assisted matrix solid-phase dispersion for the extraction and determination of multicomponent from musk by gas chromatography-mass spectrometry. J. Anal. Methods Chem. 2021, 9913055. 10.1155/2021/9913055 34422434 PMC8378966

[B62] Wang T. Yang M. Shi X. Tian S. Li Y. Xie W. (2025). Multiomics analysis provides insights into musk secretion in muskrat and musk deer. GigaScience, 14, giaf006. 10.1093/gigascience/giaf006 40036429 PMC11878540

[B47] Wang T. Zou H. Li D. Gao J. Bu Q. Wang Z. (2023). Global distribution and ecological risk assessment of synthetic musks in the environment. Environ. Pollut. 331, 121893. 10.1016/j.envpol.2023.121893 37245793

[B48] Wang Y. Sun M. Chang F. Wang J. Wang Y. Tang J. (2022). The essential differences in microbial and chemical components of musk of different qualities secreted by captive male forest musk deer (*Moschus berezovskii*). Microb. Biotechnol. 15, 1783–1794. 10.1111/1751-7915.14002 35100485 PMC9151339

[B49] Wishart D. S. (2016). Emerging applications of metabolomics in drug discovery and precision medicine. Nat. Rev. Drug Discov. 15, 473–484. 10.1038/nrd.2016.32 26965202

[B50] Wu J. Y. Wang W. (2006). The musk deer of China (in Chinese). Beijing: China Forestry Publishing House.

[B51] Xie W. Mu S. Zhong J. Zhang C. Zhang H. Wang X. (2022). Mass spectrometry imaging of lipids in the scent glands of muskrat (*Ondatra zibethicus*) in different reproductive statuses. Cells 11, 2228. 10.3390/cells11142228 35883671 PMC9322022

[B52] Xie W. Tang Z. Xu L. Zhong J. Zhang H. Han Y. (2020). Seasonal expressions of SF-1, StAR and P450scc in the scent glands of the muskrats (*Ondatra zibethicus*). J. Steroid Biochem. Mol. Biol. 204, 105766. 10.1016/j.jsbmb.2020.105766 32991988

[B53] Xu Z. Li F. Liu Q. Ma T. Feng X. Zhao G. (2024). Chemical composition and microbiota changes across musk secretion stages of forest musk deer. Front. Microbiol. 15, 1322316. 10.3389/fmicb.2024.1322316 38505545 PMC10948612

[B54] Yang Q. Meng X. Xia L. Feng ZJBC (2003). Conservation status and causes of decline of musk deer (Moschus spp.) in China. Biol. Conserv. 109, 333–342. 10.1016/s0006-3207(02)00159-3

[B55] Yeh S. Hu M. (2000). Antioxidant and pro-oxidant effects of lycopene in comparison with beta-carotene on oxidant-induced damage in Hs68 cells. J. Nutr. Biochem. 11, 548–554. 10.1016/s0955-2863(00)00117-0 11137891

[B56] Yuan N. Qin Y. Wang J. Shen L. Gao H. Xiang R. (2021). Musk secretion of endangered Alpine musk deer (*Moschus chrysogaster*): muscone content and the relationships to age, health, mating history and enclosure condition. Biologia. 76, 3761–3767. 10.1007/s11756-021-00879-7

[B57] Zhang H. L. (2022). A method for synthesizing musketone from gene-edited yeast *in vitro* . China, 1–6. Chinese invention patent.

[B58] Zhang T. Jin W. Yang S. Li Y. Zhang M. Shi M. (2021). Study of compositions of musks in different types secreted by forest musk deer (*Moschus berezovskii*). PLoS One 16, e0245677. 10.1371/journal.pone.0245677 33725016 PMC7963063

[B61] Zhang H. B. He Y. Jia G. H. Hong X. K. Wang Z. H. Ye Y. Q. (2002). Quantitative analysis for multi-components of musk by gas chromatography/mass spectrometry. Chinese Traditional Patent Medicine. 24, 868–871. 10.3969/j.issn.1001-1528.2002.11.017

[B59] Zheng C. L. Jiang G. M. Wu J. Wang J. M. Chen F. Feng D. Y. (2020). Determination and analysis of muscone content in musk produced by captive forest musk deer (in Chinese). Mod. Chin. Med. 22, 2021–2025. 10.13313/j.issn.1673-4890.20191126001

[B60] Zhou W. Qi D. Swaisgood R. R. Wang L. Jin Y. Wu Q. (2021). Symbiotic bacteria mediate volatile chemical signal synthesis in a large solitary mammal species. ISME J. 15, 2070–2080. 10.1038/s41396-021-00905-1 33568789 PMC8245644

