# Supplementary figures and images for: Elucidating metabolites and biosynthetic pathways during musk maturation: insights from forest musk deer
Source: Front Pharmacol. 2025 Apr 28;16:1503138. doi: 10.3389/fphar.2025.1503138 (PMC12066291; doi:10.3389/fphar.2025.1503138)

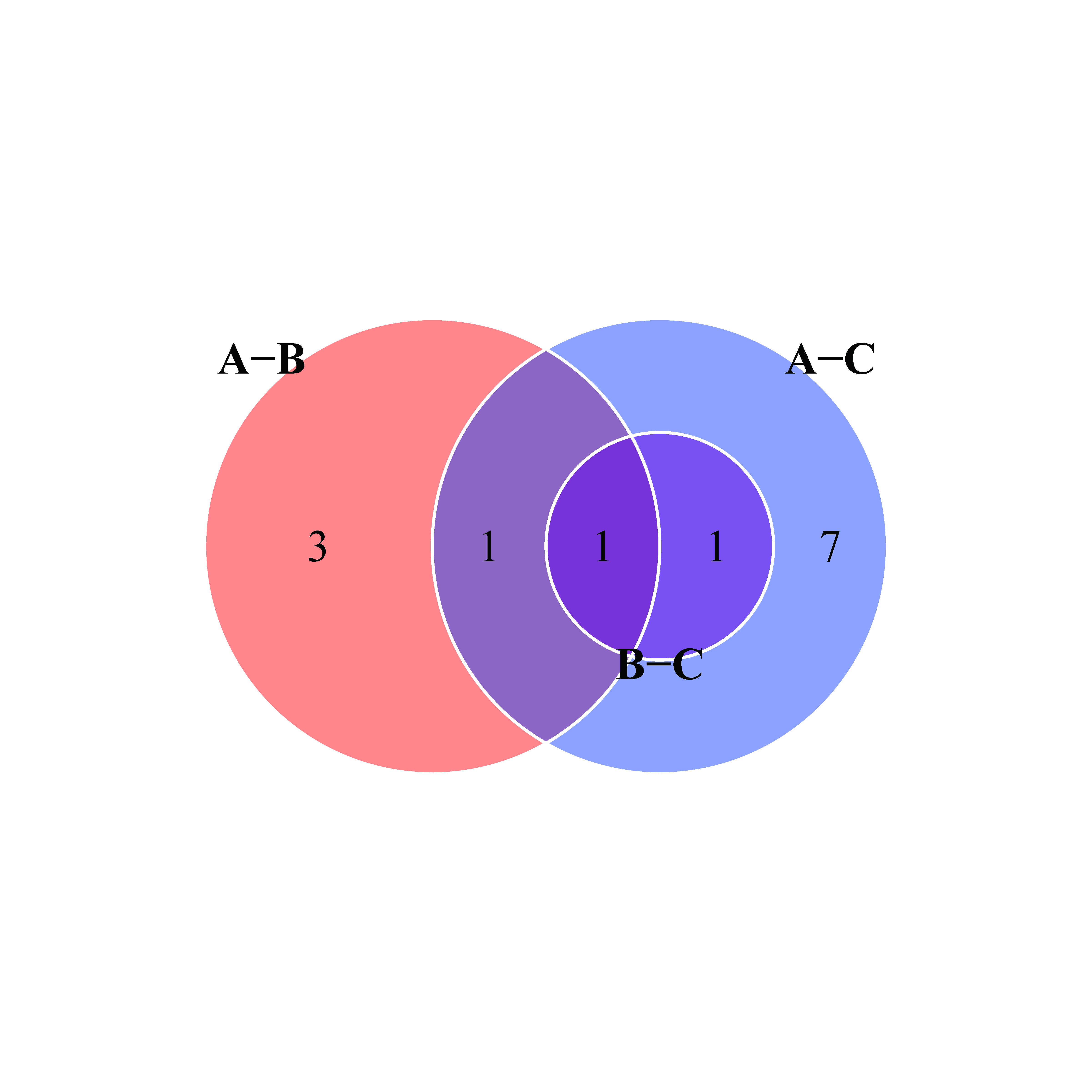

Supplement: Supplementary file 1 [file Image1.tif]
